# Fabrication and characterization of amidoxime-functionalized silica decorated with copper: a catalytic assembly for rapid reduction of dyes

**DOI:** 10.3906/kim-2007-49

**Published:** 2021-04-28

**Authors:** Ijaz Ahmed KHAN, Muhammad INAAM UL HASSAN, Hazrat HUSSAIN, Syed Mujtaba SHAH, Tariq YASIN

**Affiliations:** 1 Department of Chemistry, Quaid-i-Azam University, Islamabad Pakistan; 2 Department of Chemistry, Pakistan Institute of Engineering and Applied Sciences, Islamabad Pakistan; 3 Department of Chemistry, Women University of Azad Jammu & Kashmir, Bagh Pakistan

**Keywords:** Catalyst, composites, degradation, grafting, adsorption

## Abstract

In this study, amidoxime-functionalized silica decorated with copper (AFS-Cu) was fabricated and tested for its catalytic application. Fourier transform infrared spectroscopy, scanning electron microscopy, energy dispersive X-ray spectroscopy, and X-ray diffraction were employed to characterize its structure and morphology. The application of AFS-Cu as a catalyst for the catalytic reduction of methylene blue (MB) in aqueous media using NaBH_4_ as reductant was evaluated. The ability to reuse as well as the effect of catalyst dose and pH of solution on the catalytic activity was investigated. The reduction of MB followed pseudo-first-order kinetics and the rate constant (k) was 0.6224 min^-1^. AFS-Cu was found to be a highly effective catalyst for MB reduction reaction and can be easily recovered and reused several times with no appreciable loss of catalytic activity.

## 1. Introduction

The rapid increase in population and industrial activities is a matter of great concern. As a result, the environment of our planet deteriorates, especially the water resources. Now, water pollution has become a serious problem for human health and aquatic ecology. During the production of commercial products by industries such as textile, pharmaceutical, petroleum, paper, and pulp, a large amount of contaminated water containing both organic and inorganic materials is released [1]. The organic components of the contaminated water contain dyes and solvents which are widely used by several industries. Untreated wastewater discharge to the environment has caused many health issues and has also disturbed the aquatic ecosystem. Generally, the aqueous discharge released by many industries is hard to clean due to high toxicity, persistent and recalcitrant nature of compounds, high chemical oxygen demand, and low biodegradability [2]. 

The textile industry is the major source of dyes and about 50% of natural-fiber-based textiles are dyed. Approximately 10,000 different dyes and pigments are used for this purpose nowadays. More than 15% of these dyes are wasted during the dying process and discharged through industrial effluent [3]. The nonbiodegradable, mutagenic, teratogenetic, and carcinogenic nature of these dyes creates serious health hazards to human and aquatic organisms [4]. Therefore, it is necessary to pretreat the dye-containing effluent before discharging into the water resources.

Methylene blue (MB) is one of the most common dyes used in the textile, wood, printing, and leather industries. It is also used as a dye for histological tissue sections and photosensitizers on semiconductors etc. [5]. MB is toxic and may cause irritation, allergic problems, vomiting, diarrhea, and nausea and disturb the breathing system if inhaled or ingested [6–8]. A variety of biological and physicochemical methods have been used for the removal of dyes from wastewater. These include biosorption [9,10], biodegradation [11], adsorption [12], ultrafiltration [13], nanofiltration [14], coagulation and sedimentation [15,16], Fenton process [17], sonolysis [18], ozonation [19] etc. Recent reports showed that some of these conventional methods are not suitable for dye removal due to high cost, time-consuming processing, and stability issues [20]. Although some of these methods are versatile and useful, they all produce secondary waste products that need to be processed further due to the stable and complex aromatic structure of dyes [21]. Thus, there is a scope to develop an efficient method that is relatively cost-effective, versatile, and with no secondary waste product, i.e. sludge. 

In recent years, multistep processes have been developed for the degradation of dyes. Reductive degradation of dyes by metallic catalysts has received much attention due to high efficiency and greener root of degradation, i.e. end products are biodegradable [22]. Suvith and Philip used gold and silver nanoparticles for the degradation of MB [23]. Dobrucka reported the catalytic reduction of MB and crystal violet with platinum nanoparticles [24]. Li et al. used Pd/Fe3O4-PEI-RGO nanohybrids for catalytic degradation of MB [25]. Ai and Jiang reported the alginate hydrogel-capped silver nanoparticle-assisted catalytic reduction of 4-nitrophenol by NaBH_4_ [26]. Most of the reported work related to the catalytic reductive degradation of dyes was carried out with precious and noble metals (e.g., gold, silver, platinum, and palladium). The high cost and less availability of these metals make them less feasible for these applications [27]. Moreover, these nanoparticles are unstable, prone to agglomerate, and cannot be easily separated from the reaction medium [28]. To overcome these problems, a variety of solid supports such as graphene, titanium oxide, eggshell, and iron oxide have been used for the development of catalysts [29]. 

Thus, there is a scope to develop an efficient material that is relatively cost-effective, versatile, and with no secondary waste product. Radiation grafting is established as a useful technique to fabricate functional materials without affecting the mechanical and chemical properties of the base material. The grafting process can be initiated by high energy radiation such as gamma rays and electron beams via mutual or preirradiation method [30,31]. A variety of functional materials fabricated by radiation grafting of different monomers onto different base materials such as mesoporous silica [32], clay [33], and polypropylene [34] are reported in the literature.

The present study was focused to develop a stable, cost-effective, and efficient catalyst for the reduction of dyes, which can easily be separated from the reaction medium by simple filtration. Amidoxime-functionalized silica (AFS) was used as support to develop the copper-loaded catalyst in this study. MB was chosen as a model dye and its heterogeneous degradation was carried out with NaBH_4_ in the presence of the developed catalyst. The effect of different operational parameters as pH, catalyst dose, and time was also investigated. 

## 2. Materials and methods

### 2.1. Materials 

For the present study, all the chemicals and reagents were of analytical grade and used as received. Isopropanol, vinyltriethoxysilane (VTES), copper sulfate, acrylonitrile, MB, sodium carbonate, sodium borohydride (NaBH_4_), and hydroxylamine hydrochloride were purchased from Sigma Aldrich (supplied by Chem Tech Pakistan, Karachi, Pakistan). Silica was purchased from Amicon Corporation (Massachusetts, USA), having a particle size of 35–70 µm, a specific surface area of 250 m2/g, and a pore diameter of 25 nm.

### 2.2. Method of preparation of the catalyst

Amidoxime-functionalized silica was prepared following the procedure reported in our previous study [32]. Briefly, the silica was first modified with VTES, and then the modified silica was grafted with acrylonitrile by radiation-induced grafting in an inert atmosphere by Co-60 irradiator, at Nuclear Institute for Food and Agriculture, Peshawar at a dose rate of 4.7 kGy/h. The nitrile groups of the grafted acrylonitrile were converted into amidoxime by treating with hydroxylamine solution. Amidoxime-functionalized silica (AFS) was loaded with copper by shaking an appropriate amount of oven-dried AFS in 100 mL of copper sulfate solution having pH of 5 and 500 ppm concentration. The continuous shaking was carried at a rate of 200 rpm for 2 h. After the shaking was complete, the copper-loaded AFS particles were separated from the reaction medium by filtration, washed with distilled water several times to remove the loosely bonded copper, and dried in oven at 60 °C. The oven-dried sample was notated as AFS-Cu and used in catalytic reduction of MB. Quantitatively, the amount of Cu in AFS-Cu was estimated to be ~200 mg of Cu per gram of AFS, which was calculated gravimetrically by measuring the weight of the composite before and after the copper loading.

### 2.3. Catalyst characterization

The morphology of the samples was investigated by scanning electron microscopy (SEM) using a Tescan, MIRA-3 field emission scanning electron microscope. Samples used in the SEM tests were deposited directly onto stubs and examined without further treatment. Energy dispersive X-ray spectroscopy (EDX) analysis was carried out to show the presence of elements Si, N, O, and Cu, etc. The crystalline structure of AFS-Cu was examined using a Panalytical Xpert Pro X-ray Diffractometer equipped with Cu Kα (1.5418 Å) radiation source, a Ni filter at a setting of 40 kV/30 mA, and a scintillation counter detector. The data was recorded over 20°–80°, 2 θ range. Fourier transform infrared (FTIR) spectroscopy was carried out using a Nicolet 6700 FTIR spectrophotometer from Thermo Electron Corporation, (Waltham, USA), using the attenuated reflectance mode. FTIR spectra were recorded in the range from 4000 to 400 cm^-1^. 

### 2.4. Catalytic reductive degradation of dye by AFS-Cu

To investigate the catalytic activity of the synthesized AFS-Cu catalyst, catalytic reductive degradation of MB with NaBH_4_ was carried in the presence of AFS-Cu. In a typical reduction experiment, MB aqueous solution (5 mL) was mixed with AFS-Cu (20 mg). After that, a freshly prepared solution of NaBH_4_ (1 mL, 0.1 M) was added to the above mixture. The progress in dye reduction was monitored using a UV-visible spectrometer. A blank experiment without AFS-Cu was also carried out. The effects of different reaction parameters, such as pH, catalyst dose, and time were also studied. Furthermore, by employing atomic absorption spectroscopy, it was confirmed that copper does not leach out into the solution. 

## 3. Results and discussions

### 3.1. Catalyst characterization 

**Figure 1:**
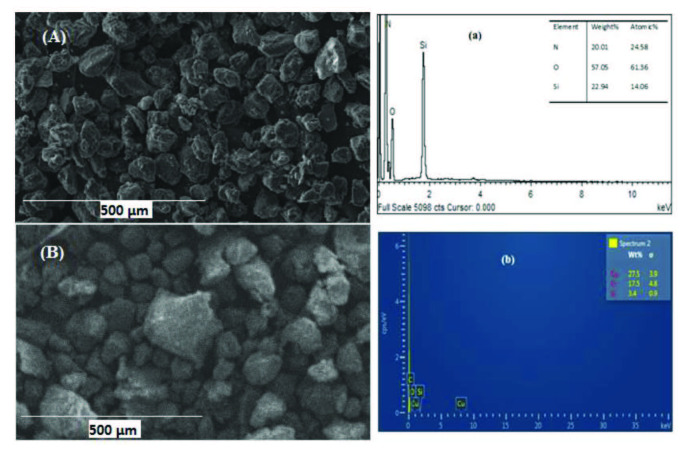
SEM images of AFS (A), AFS-Cu catalyst (B), and EDX of AFS (a), AFS-Cu catalyst (b).

SEM micrographs of AFS and AFS-Cu were displayed in Figures 1A and 1B, respectively. SEM image of AFS shows that the particles are of irregular shape and rough surface which might be due to grafting. The copper-loaded particles show some agglomerations and changed morphology as shown in Figure 1B. However, no major change in the overall morphology of the particles was observed during copper loading. 

EDX analysis of AFS shows the presence of N, O, and Si as shown in Figure 1a. This correlates with the amidoxime functional groups present on silica. AFS-Cu shows a high weight (%) of copper besides these elements as shown in Figure 1b, which confirms the copper loading. The proposed mechanism of interaction of Cu^2+^ ions with amidoxime group is reported in our previous study [32].

**Figure 2:**
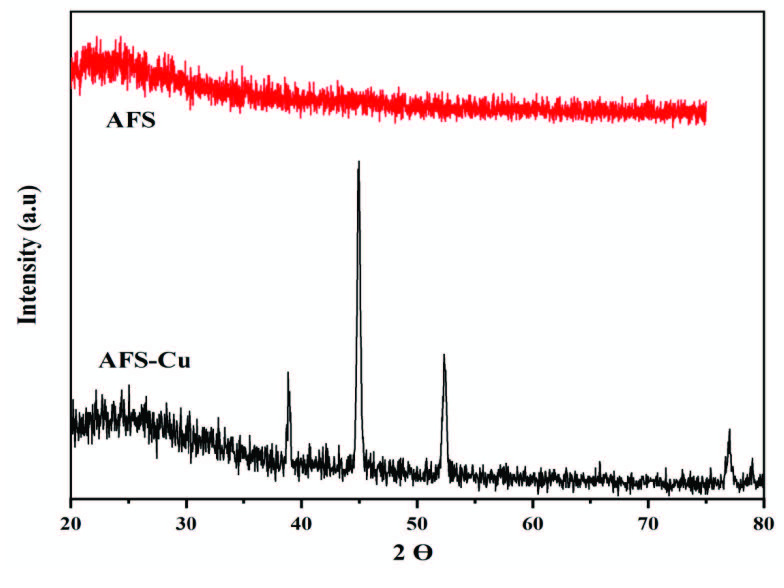
XRD patterns of AFS and AFS-Cu catalyst.

The XRD studies were used to explore the crystal structure of AFS and AFS-Cu, and the results are depicted in Figure 2. XRD of AFS shows a single broad band centered at 2θ *= *22°. This broad band was due to the amorphous nature of silica-bearing amidoxime groups in AFS. XRD diffractogram of AFS-Cu showed intense peaks at 2θ values of 38.86°, 44.97°, 52.28°, and 77.03°. The peak at 38.86° indicates that a small but detectable amount of copper was also present in the form of oxide. The peaks at 44.97°, 52.28°, and 77.03° correspond to the miller indices (111), (200), and (220) of face centered cubic structure of Cu crystals, respectively. Similar results have been reported in the literature [35–41].

**Figure 3:**
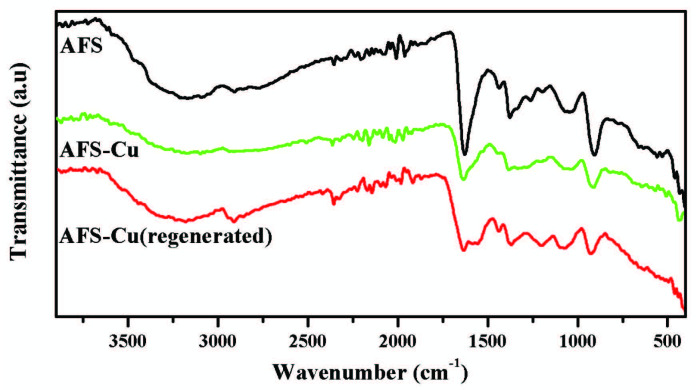
FTIR spectra of AFS, AFS-Cu catalyst, and AFS-Cu (regenerated) catalyst.

Figure 3 shows the FTIR spectra of AFS, AFS-Cu, and AFS-Cu (regenerated). AFS shows a broad band at 1000–1150 cm^-1^ assigned to Si–O–Si– stretch, and a small peak at 1256 cm^-1^ which was the –Si–CH_2_ stretch. In addition, the peaks at 3000–3400 cm^-1^ can be assigned to –OH, at 2800–3000 cm^-1^ to –CH, at 1640 cm^-1^ to -C=N and at 920 cm^-1^ to the >N–O stretch, which are the characteristic functionalities present in amidoxime functional groups [42]. The AFS-Cu spectrum shows all these peaks with reduced intensity, indicating that these functionalities are involved in binding with copper. The FTIR spectrum of the regenerated catalyst after the third cycle shows an almost similar spectrum as that of fresh AFS-Cu and no appreciable change was observed indicating that the catalyst retains its structural stability during use. 

### 3.2. Catalytic reductive degradation of dye by AFS-Cu

**Figure 4:**
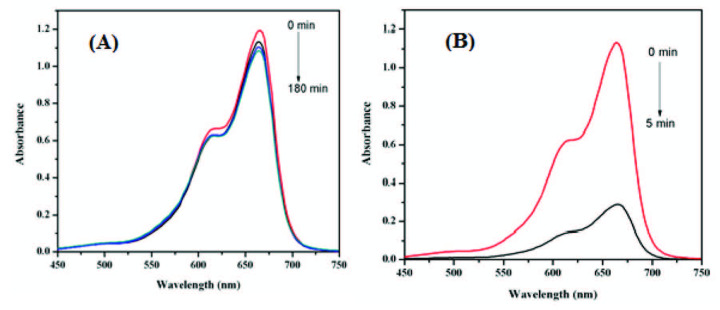
Reductive degradation of MB by NaBH4 at room temperature (A) in the absence of AFS-Cu catalyst, (B) in the presence of AFS-Cu catalyst.

The catalytic activity of the AFS-Cu catalyst was monitored using MB as a model dye. The progress in catalytic reduction was examined by a decrease in absorbance at 664 nm using UV-Visible spectroscopy. The spectra of the reduction of MB with NaBH_4_ in the presence and absence of catalyst are depicted in Figure 4. Figure 4A shows the spectra of reduction of MB in the absence of AFS-Cu catalyst up to 180 min. As expected, the reduction process in the absence of catalyst was very slow with little degradation up to 180 min. 

**Figure 5:**
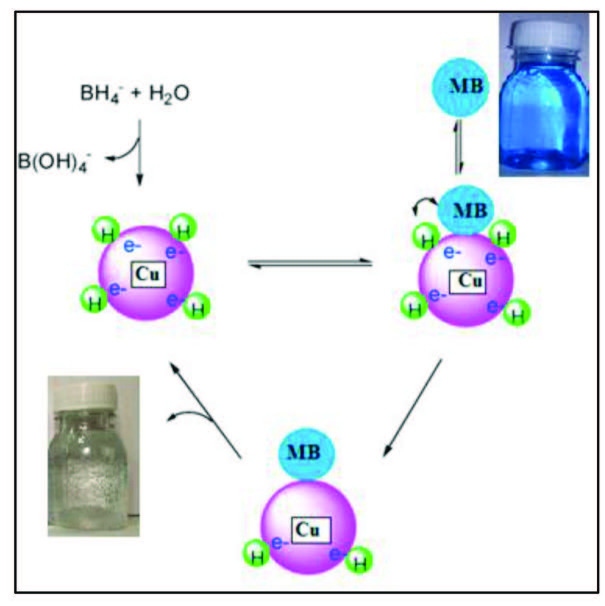
Proposed mechanism for MB degradation by AFS-Cu catalyst.

Figure 4B depicts the spectra of reduction of MB in the presence of AFS-Cu catalyst. These results demonstrate that the AFS-Cu catalyst is necessary for the fast reduction of MB in this reaction. The catalyst serves as an electron mediator between BH_4_^-^ and MB, electron transfer takes place via Cu present on the catalyst [29]. The reduction process typically proceeded in two stages: (i) BH_4_^-^ diffusion and adsorption of dye on the catalyst surface and (ii) transfer of electron from BH_4_^-^ to the dye mediated by the catalyst. The dye will then be reduced on the catalyst surface and desorbed from the surface to continue the reaction as shown in Figure 5.

### 3.3. Effect of catalyst loading on the reduction of MB

**Figure 6:**
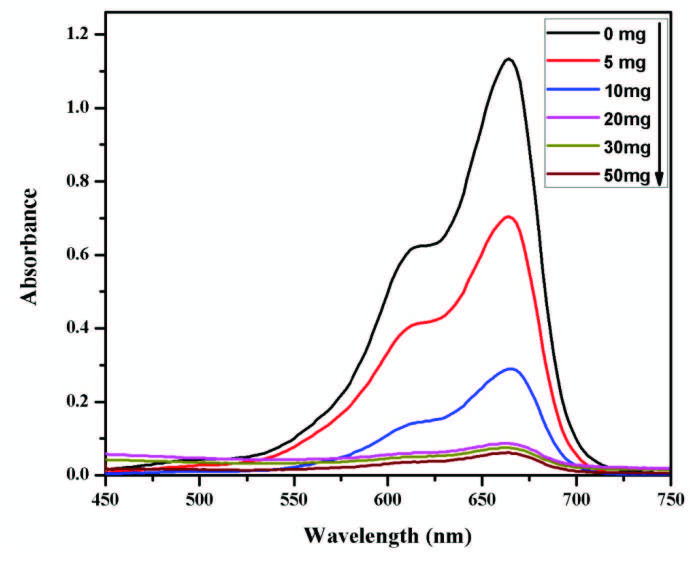
Effect of catalyst dose on MB degradation (MB: 5 mL, 5 ppm and NaBH4: 1 mL, 0.1 M, time: 4 min) at room temperature.

It has been reported that the amount of catalyst also influences the rate of reaction [41]. To investigate this effect, a varying amount of catalyst ranging from 5 to 50 mg was used and the obtained results are shown in Figure 6. This figure depicts the UV-visible spectra of MB degradation at different catalyst doses.

These results showed that a rapid increase in degradation was observed when the catalyst amount was increased from 5 mg to 20 mg. This rapid increase showed that the effective concentration of the catalyst is 20 mg above this concentration, a very little change in degradation was observed. This behavior might be since once the catalyst amount reached a certain point (20 mg in this study), the adsorption of dye on the catalyst surface reached an optimum value and 20 mg of catalyst was used in further studies. 

### 3.4. Effect of pH on catalytic reduction of MB

**Figure 7:**
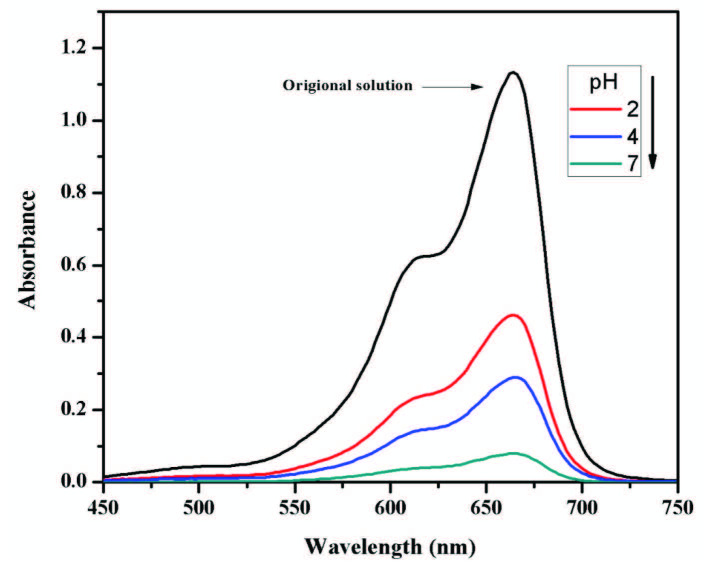
Effect of pH on MB degradation (AFS-Cu: 20 mg; MB: 5 mL, 5 ppm and NaBH4: 1 mL, 0.1 M, time: 4 min) at room temperature.

The pH of the solution is an important factor that significantly affects the degradation process. The effect of pH on MB degradation was studied at different pH values of the solution in the range of 2–7 and the results are shown in Figure 7. 

The results show that the change in pH from acidic to neutral results in an increase in the degradation process. This phenomenon at low pH might be due to the protonation of catalyst and dye which led to a strong repulsion between them. As a result, less degradation was observed at this pH. On the other hand, the increase in pH led to the deprotonation of dyes caused an increase in degradation. Similar results were reported by Mosleh et al. using CuO/Cu_2_O/Cu nanoparticles for the photocatalytic degradation of dyes [35]. Therefore, the optimum value of pH was adjusted at 7.

### 3.5. Kinetic study of catalytic reduction of MB

**Figure 8:**
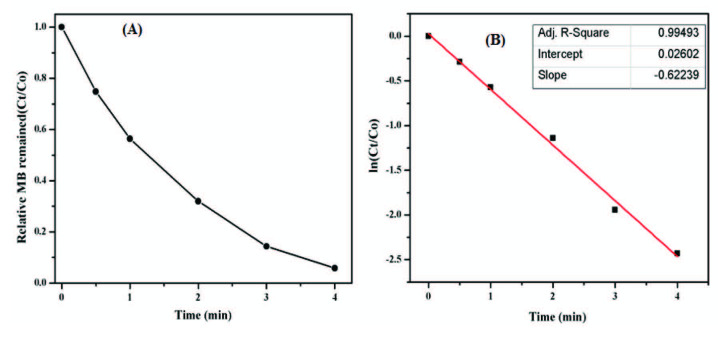
(A) Time-dependent change in MB solution (At/Ao) in the presence of NaBH4 and AFS-Cu catalyst, (B) is a linear relationship of ln (At/Ao) as a function of time for MB solution.

The kinetics of the catalytic reductive reaction of MB by NaBH_4_ in the presence of AFS-Cu was monitored at different time intervals. The plot of At/Ao versus reaction time shows a profile of exponential nature, which also indicated the reduction reaction follows the pseudo-first-order kinetics as shown in Figure 8A. The rate constant (k) of the reaction was calculated by using the following equation:

Ln(CtC0)=ln(AtA0)=-kt

where C_0_ is the initial concentration and C_t_ is the concentration of MB at time t, and A_t_ and A_0_ are the corresponding absorbance values, respectively. 

Figure 8B depicted the plot of ln (A_t_/A_0_) vs. reaction time (t) and slope determines the rate constant for the reaction. The plot presented a good linear relationship with a correlation coefficient (R2) of 0.9949 and the calculated rate constant was 0.6224 min^-1^.

### 3.6. Recovery and reusability of the catalyst

**Figure 9:**
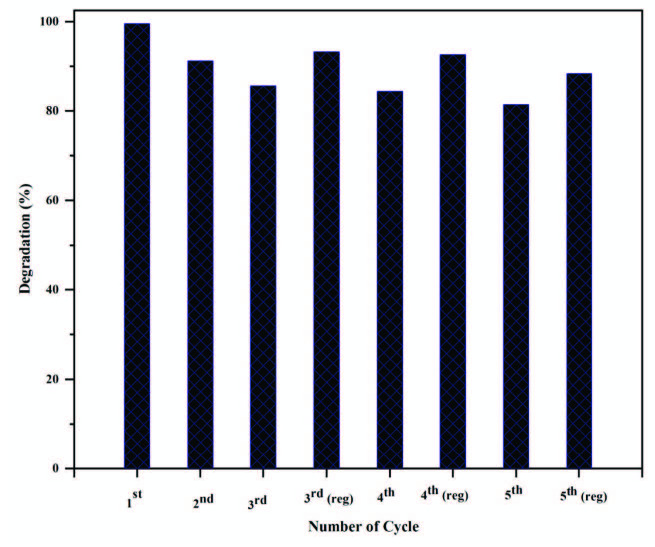
Comparative results of MB degradation from the 1st to 3rd cycle without regeneration and regenerated AFS-Cu catalyst after that up to the 5th cycle.

The easy recovery of the catalyst (AFS-Cu) from the reaction mixture by simple filtration is an important feature that makes it suitable for industrial applications. The micrometer-size particles can be easily recovered by filtration and reused after simple washing with deionized water and dried in oven at 50 °C. Figure 9 depicts the comparative results of catalytic reduction of methylene blue by AFS-Cu. It can be seen from the figure that the recovered catalyst can be used three times with a slight decrease in the activity of the catalyst. After the 3rd cycle, the recovered catalyst was washed each time up to the 5th cycle with alcoholic water (10%) and then with an excess of deionized water.

This regenerated catalyst was reused. The results demonstrate that the regenerated catalyst regains its activity almost equal to the fresh catalyst, and only a slight loss in activity was observed up to the 5th cycle. The catalytic activity of AFS-Cu is compared with other reported catalysts and the results are shown in Table. These results indicate that AFS-Cu can serve as a promising catalyst for the degradation of dyes (MB). 

**Table:** Comparison of results for catalytic degradation of MB with a variety of catalysts.

Catalyst	Time (min)	Reference
AFS-CuAg NPs on silica spheresPorous Cu microspheresBiosynthesized Ag nanoparticlesSiNWAs-CuCrO(OH) nanoparticlesCuNFs	47.588103085	Present study[42][43][44][45][46][47]

## 4. Conclusion

Amidoxime-functionalized silica decorated with copper was successfully synthesized and characterized. The synthesized material was used as a catalyst in the heterogeneous catalytic reductive degradation of MB in the presence of NaBH_4_. The effect of different operational parameters was optimized and the best results were obtained at neutral pH and 20 mg of catalyst dose. The catalytic reductive degradation reaction followed pseudo-first-order kinetics with a rate constant of 0.6224 min^-1^. The results demonstrated that AFS-Cu is an efficient and economic catalyst for the degradation of dyes as copper is a less expensive metal as compared to the other metals (Ag, Au, and Pd) used in most earlier studies. The catalyst has excellent stability and is used for 5 cycles with a slight loss in activity. Moreover, this catalyst can be easily recovered by simple filtration and regenerated by washing with alcoholic water. The obtained results show that AFS-Cu catalyst has advantages over other catalysts in terms of cost, stability, recovery, and reusability, etc.
